# Editorial: STI awareness day: diagnosis and control of STIs in LMICs

**DOI:** 10.3389/frph.2024.1415433

**Published:** 2024-04-25

**Authors:** Ravesh Singh, Nathlee Abbai, Teke R. Apalata

**Affiliations:** ^1^ Department of Medical Microbiology, School of Laboratory Medicine and Medical Sciences, College of Health Sciences, University of KwaZulu-Natal, Durban, South Africa; ^2^ National Health Laboratory Service, Durban, South Africa; ^3^ School of Clinical Medicine Laboratory, College of Health Science, University of KwaZulu-Natal, Durban, South Africa; ^4^ National Health Laboratory Service, Nelson Mandela Academic Hospital, Mthatha, South Africa; ^5^ Department of Laboratory Medicine and Pathology, Faculty of Health Sciences, Walter Sisulu University, Mthatha, South Africa

**Keywords:** STIs, awareness, diagnosis, control, LMICs, testing, stigma, education

**Editorial on the Research Topic** STI awareness day: diagnosis and control of STIs in LMICs

Sexually transmitted infections (STIs) remain a significant public health concern globally, disproportionately impacting low- and middle-income countries (LMICs). While STI Awareness Day serves as a crucial reminder for all populations, the challenges faced by LMICs require specific focus and tailored solutions. This editorial piece delves into the complexities of STI diagnosis and control in LMICs, drawing upon recent research to illuminate the path forward.

One of the major hurdles for effective STI management in LMICs is illustrated by the case report titled “Chronic epididymitis due to *Chlamydia trachomatis* LGV-L2 in an HIV-negative heterosexual patient: a case report”. This report described an unusual case of chronic inflammation in the male reproductive tract caused by a specific strain of *Chlamydia*. This highlighted the potential for atypical presentations and the need for robust diagnostic tools to ensure accurate identification of all STI causes Paira et al.

Accurate diagnosis is essential for timely and appropriate treatment. However, limited access to healthcare facilities and diagnostic tests in LMICs often leads to delayed diagnoses and potential complications. The study “Biobehavioral survey using time location sampling among female sex workers living in Ghana in 2020” played a vital role in understanding the specific demographics and risk factors associated with STIs in vulnerable populations. This study, conducted among female sex workers (FSWs) in Ghana, not only shed light on their sexual behaviors but also revealed the prevalence of STIs within this group. Such data allows for targeted interventions and improved access to STI testing and treatment services for high-risk populations Dery et al.

The consequences of undiagnosed and untreated STIs can be severe, particularly for women. The systematic review titled “The association between adverse pregnancy outcomes and non-viral genital pathogens among women living in sub-Saharan Africa: a systematic review” underscores this concern. The review highlighted the link between non-viral STIs, such as bacterial vaginosis and trichomoniasis, and adverse pregnancy outcomes, including premature birth, low birth weight, and neonatal mortality. Emphasizing the importance of STI control for maternal and child health in sub-Saharan Africa, this research underscores the need for comprehensive antenatal care that includes STI screening and treatment Gamberini et al.

Beyond diagnosis and treatment, strengthening prevention strategies is crucial for curbing the spread of STIs in LMICs. Research such as “HIV counseling, testing, and test result receipt among East African women of reproductive age using recent national health surveys” provided valuable insights into existing gaps in access to essential services. This study which focused on HIV testing among women in East Africa, highlighted the need for improved access to comprehensive sexual and reproductive health services, including STI testing and counseling Terefe.

The research presented underscores the need for a multifaceted approach to address the challenges of STI diagnosis and control in LMICs. Here are some key areas for action:

Strengthening healthcare infrastructure: Expanding access to healthcare facilities and diagnostic tests in remote areas is critical. This includes investing in laboratory equipment, personnel training, and point-of-care testing technologies. Point-of-care tests, which provide rapid results at the point of care without the need for a laboratory, can significantly improve access to STI diagnosis in resource-limited settings (1). Examples of successful point-of-care tests include rapid diagnostic tests (RDTs) for syphilis and HIV, which can be used in resource-limited settings (2).

Investing in community outreach: Educational campaigns and community engagement initiatives are crucial to raise awareness about STIs, their symptoms, and the importance of seeking testing and treatment. Tailoring messages to specific populations, including Female Sex Workers and young people, is essential. Peer education programs, utilizing trained community members, can be particularly effective in reaching marginalized populations and addressing stigma associated with STIs (3).

Promoting healthy sexual behavior: Comprehensive sexual education programs should be implemented to empower individuals to make informed choices and protect themselves from STIs. These programs should address topics such as abstinence, condom use, negotiation skills, and healthy relationships. School-based programs and community workshops can provide a platform for delivering essential sexual health information to young people (4).

Addressing social determinants of health: Poverty, gender inequality, and limited access to education all contribute to the spread of STIs. Empowering women economically and ensuring access to education can help them negotiate safer sex practices and reduce their vulnerability to STIs (5).

Integration with existing programs: Integrating STI prevention, testing, and treatment services with existing healthcare programs, such as those for HIV/AIDS or maternal health, can improve accessibility to diagnostic tests and efficiency of treatments.
